# Promiscuous Binding of Invariant Chain-Derived CLIP Peptide to Distinct HLA-I Molecules Revealed in Leukemic Cells

**DOI:** 10.1371/journal.pone.0034649

**Published:** 2012-04-26

**Authors:** Marvin M. van Luijn, Arjan A. van de Loosdrecht, Margit H. Lampen, Peter A. van Veelen, Adri Zevenbergen, Michel G. D. Kester, Arnoud H. de Ru, Gert J. Ossenkoppele, Thorbald van Hall, S. Marieke van Ham

**Affiliations:** 1 Department of Hematology, Cancer Center Amsterdam, VU Institute for Cancer and Immunology, VU University Medical Center, Amsterdam, The Netherlands; 2 Sanquin Research and Landsteiner Laboratory, Department of Immunopathology, Academic Medical Center, University of Amsterdam, Amsterdam, The Netherlands; 3 Department of Clinical Oncology, Leiden University Medical Center, Leiden, The Netherlands; 4 Department of Immunohematology and Blood Transfusion, Leiden University Medical Center, Leiden, The Netherlands; 5 Laboratory of Experimental Hematology, Department of Hematology, Leiden University Medical Center, Leiden, The Netherlands; Albert Einstein Institute for Research and Education, Brazil

## Abstract

Antigen presentation by HLA class I (HLA-I) and HLA class II (HLA-II) complexes is achieved by proteins that are specific for their respective processing pathway. The invariant chain (Ii)-derived peptide CLIP is required for HLA-II-mediated antigen presentation by stabilizing HLA-II molecules before antigen loading through transient and promiscuous binding to different HLA-II peptide grooves. Here, we demonstrate alternative binding of CLIP to surface HLA-I molecules on leukemic cells. In HLA-II-negative AML cells, we found plasma membrane display of the CLIP peptide. Silencing Ii in AML cells resulted in reduced HLA-I cell surface display, which indicated a direct role of CLIP in the HLA-I antigen presentation pathway. In HLA-I-specific peptide eluates from B-LCLs, five Ii-derived peptides were identified, of which two were from the CLIP region. *In vitro* peptide binding assays strikingly revealed that the eluted CLIP peptide RMATPLLMQALPM efficiently bound to four distinct HLA-I supertypes (-A2, -B7, -A3, -B40). Furthermore, shorter length variants of this CLIP peptide also bound to these four supertypes, although *in silico* algorithms only predicted binding to HLA-A2 or -B7. Immunization of HLA-A2 transgenic mice with these peptides did not induce CTL responses. Together these data show a remarkable promiscuity of CLIP for binding to a wide variety of HLA-I molecules. The found participation of CLIP in the HLA-I antigen presentation pathway could reflect an aberrant mechanism in leukemic cells, but might also lead to elucidation of novel processing pathways or immune escape mechanisms.

## Introduction

In immune surveillance against invading pathogens and tumor cells, antigen processing and presentation by HLA molecules is essential for induction of potent T cell-mediated immunity.

Classically, exogenously derived antigens, such as bacterial components, are processed in the endosomal/lysosomal system for loading onto HLA class II (HLA-II) complexes. After synthesis in the endoplasmic reticulum (ER), the HLA-II heterodimer binds to the invariant chain (Ii) for transport to late endosomes [1]. Here, Ii is cleaved until only a small fragment, the class II-associated invariant chain peptide (CLIP) remains bound to the class II peptide-binding groove [2]. In MHC class II loading compartments (MIICs), CLIP is exchanged for an antigenic peptide with aid of HLA-DM [3], [4], and HLA-II/peptide complexes are exported to the plasma membrane and presented to CD4^+^ T cells. In tumor cells that have APC function, efficient processing of endogenous, potentially tumor-associated antigens (TAAs) is pivotal for T cell priming of and/or recognition by specific effector T cells. We and others previously showed that such endogenous antigen presentation can also involve HLA-II complexes [5], [6]. Ii silencing in certain tumor cells downmodulates CLIP, but not HLA-II expression levels [7] and results in increased presentation of endogenous antigens and tumor-specific CD4^+^ T cell activation [5], [8]. These studies contradict with the proposed requirement of Ii for HLA-II stabilization and transport [9], but agree with its function in preventing binding of endogenous peptides to HLA-II complexes in the ER [10].

For HLA-I antigen presentation, endogenous proteins, *e.g.* tumor- and virus-associated proteins, are normally degraded by the cytoplasmic proteasome followed by translocation of peptides into the ER via the transporter associated with antigen processing (TAP) molecule. Here, peptides with the appropriate binding motif associate with newly formed HLA class I (HLA-I) heavy chain/β2m heterodimers and are transported to the plasma membrane for presentation to CTLs (reviewed in [11]). Professional APCs, including macrophages, dendritic cells (DCs) and B cells, have well-equipped machinery to detect, internalize and process exogenous antigens. These antigens are processed for HLA-II-mediated presentation, but can also be routed for presentation by HLA-I, resulting in cross-priming of CTLs (reviewed in [12]). Two general routes for this so-called cross-presentation have been described: exogenous antigens are degraded and directly loaded onto HLA-I molecules in the endo-lysosomal pathway [13] or, alternatively, gain access to the cytoplasm for proteasome-dependent processing and are directed either back into endosomes or the ER via TAP [14], [15]. The precise mechanism by which HLA-I molecules may enter the endo-lysosomal pathway is poorly defined.

Ii is a type II transmembrane protein that exists in different isoforms and contains one or more internal targeting signals for specific transport of newly synthesized HLA-II complexes to the MIICs [16], [17]. In addition to its role in HLA-II transport, the role of Ii as chaperone seems to be more versatile. Ii binds to the actin-based motor protein myosin-II to negatively affect DC migration [18], to adhesion molecule CD44 to activate T cells [19] as well as to costimulatory molecule CD70 for targeting to the MIICs [20]. In the present study, we show an accessory role for Ii and CLIP in HLA-I processing and antigen presentation by leukemic cells.

## Methods

### Patient Material

Bone marrow samples from nine newly diagnosed acute myeloid leukemia (AML) patients were collected after obtaining written informed consent and according to the Declaration of Helsinki. This was approved by the review board (‘Medisch Ethische Toetsingscommissie, METc’) of the VU University Medical Center, Amsterdam, The Netherlands. Classification of acute promyelocytic leukemia (APL) was based on standard genetic and molecular detection of t(15;17), as part of routine diagnostic procedures at our department. HLA-DR-negative AML patients contained high numbers of myeloid cells (>80% of the total WBC count), which were defined as CD45^dim^/SSC^low-int^ by flow cytometry. Mononuclear cells were isolated using Ficoll-PaquePLUS (Amersham Biosciences, Freiburg, Germany) and directly used for protein analysis or cryopreserved in liquid nitrogen.

### Cell Lines and Culturing

Human leukemic cell lines KG-1, THP-1 and Kasumi-1 were purchased from the American Type Culture Collection (ATTC). KG-1 cells were maintained in IMDM (Gibco, Paisley, UK) supplemented with 20% FBS (Greiner, Alphen a/d Rijn, The Netherlands), 25 mM Hepes (Sigma-Aldrich, St Louis, MO, USA), 1% L-glutamine and 50 µM 2-ME (both Gibco). THP-1 and Kasumi-1 were cultured in RPMI 1640 medium (Gibco) containing 10% and 15% FBS, respectively. For peptide elutions, Epstein-Barr virus (EBV)-transformed B-lymphoblastoid cell lines (B-LCLs) were generated from peripheral blood mononuclear cells (PBMC) from healthy blood donors or patients. This was performed in approval with the Leiden University Medical Center review board. EBV-transformed B-LCL JY, PHEB and 5544 were cultured in IMDM containing 8% FBS, 100 IU/ml penicillin and 2 mM L-glutamine. For the competition-based peptide binding assays, B-LCLs expressing HLA-I alleles of interest were a generous gift from Dr. J. Kessler (Leiden University Medical Center; [21]). T2 cells were obtained from Dr. P. Cresswell (Yale University School of Medicine, New Haven, CT, USA).

### Antibodies and Immunofluorescence Stainings

We used the following mouse anti-human monoclonal antibodies (MoAbs): PE-labeled anti-CLIP (clone cerCLIP.1; Santa Cruz Biotechnology, Santa Cruz, CA, USA) and anti-HLA-ABC (clone W6/32; Dako, Glostrup, Denmark); FITC-labeled CD74, anti-HLA-DR (clone L243) and anti-HLA-DRPQ; PerCP-labeled CD45 (all BD Biosciences, San Jose, CA, USA). Anti-Ii MoAb (clone PIN1.1) was kindly provided by Dr. P. Cresswell. For stainings of murine cells, PE-labeled CD8 (clone 53–6.7; Biolegend, San Diego, CA, USA) and APC-labeled anti-IFN-γ (clone XMG1.2; BD Biosciences) antibodies were used.

For immunofluorescence, cells were incubated with 10% human γ-globulin (60 mg/ml; Sanquin, Amsterdam, The Netherlands) before the MoAb of interest was added. Moabs were added during 15 min at room temperature (RT). Intracellular staining for Ii was performed on cells fixed with PBS 1% paraformaldehyde and permeabilized with PBS 0.1% saponin (Sigma-Aldrich). After incubation with PIN1.1 for 30 min at RT, cells were stained for 20 min with a secondary, PE-labeled rabbit anti-mouse (RAM) IgG (Dako). Each incubation step was followed by two washing steps in PBS with 0.1% HSA and 0.05% sodium azide. We used a FACSCalibur flow cytometer and CellQuestPro software (BD Biosciences) or Flowjo software (Treestar Inc, Ashland, OR, USA) to analyze the percentage of positive cells. Leukemic cells of AML patients were defined by CD45^dim^/SSC^low^ expression.

### Co-immunoprecipitations and Western Blotting

See File S1 in the Supporting Information section.

### Retrovirus Production and Transduction

Ii expression was silenced using the retroviral pSIREN-RetroQ vector (Clontech, Palo Alto, CA, USA) consisting of a puromycin resistance gene together with a cloned Ii siRNA insert (sequence no. 53; [22]) Retrovirus, a kind gift from Dr. S. Ostrand-Rosenberg (University of Maryland, Baltimore, USA), was produced as reported [23]. For retroviral transduction, 1×10^6^ cells/ml were cultured until 40% confluency. Following washing with PBS, cells were resuspended in DMEM (Gibco) containing 10% FBS, 10 mM Hepes and polybrene, followed by the drop-wise addition of retroviral supernatant to attain a polybrene concentration of 4 µg/ml. Cells were incubated at 37°C for 6 h, washed three times with excess PBS and kept in culture medium for three days. Subsequently, 0.5 µg/ml puromycin was added and increased gradually during a period of two weeks to a final concentration of 1.0 µg/ml in order to select Ii siRNA-transduced cells. Ii expression in transduced cells was checked by flow cytometric analysis. The process of siRNA formation and retroviral transduction itself was validated not to affect Ii expression [6], [22].

### HLA Class I Peptide Isolation, HPLC and Mass Spectrometry

HLA-I/peptide complexes were purified from >10^10^ EBV-transformed B-LCLs by affinity chromatography using protein A beads (GE Healthcare) covalently bound to MoAbs against HLA-I (clone W6/32; used for B-LCL JY) or HLA-A2 (clone BB7.2; [24]; used for B-LCL PHEB and 5544). Peptides were eluted from isolated HLA-I molecules and separated from class I heavy chains and β2m by passage through Centriprep filtration units with a 10 kD, and the complex peptide pool was fractionated on a 15 cm×200 µm RP-C18 (Reprosil-Pur C18-AQ 3 µm) column, packed in house. The gradient was run from 0% to 50% solvent B (10/90/0.1 v/v/v water/acetonitrile/formic acid) in 45 min.

Peptide fractions from the first dimension separation were reduced to near dryness and resuspended in 95/3/0.1 v/v/v water/acetonitrile/formic acid. These resuspended fractions were analyzed by on-line nano-HPLC mass spectrometry using a system earlier described [25]. Fractions were injected onto a precolumn (100 µm×15 mm; Reprosil-Pur C18-AQ 3 µm, 5 µm, Phenomenex) and eluted via an analytical nano-HPLC column (15 cm×50 µm; Reprosil-Pur C18-AQ 3 µm). The gradient was run from 0% to 50% solvent B (10/90/0.1 v/v/v water/acetonitrile/formic acid) in 90 min. The nano-HPLC column was drawn to a tip of approximately 5 µm and acted as the electrospray needle of the MS source.

For mass spectrometry, we used a LTQ-FT Ultra mass spectrometer (Thermo, Bremen, Germany) that was operated in data-dependent mode, automatically switching between MS and MS/MS acquisition. Full scan mass spectra were acquired in the Fourier-transform ion cyclotron resonance (FT-ICR) with a resolution (m/Δm at full width half maximum) of 25,000 at a target value of 5,000,000. The two most intense ions were then isolated for accurate mass measurements by a selected ion monitoring scan in FT-ICR with a resolution of 50,000 at a target accumulation value of 50,000. The selected ions were then fragmented in the linear ion trap using collision-induced dissociation at a target value of 10,000. In a post analysis process, raw data were converted to peak lists using Bioworks Browser software, Version 3.1. For peptide identification, MS/MS data were submitted to the human IPI database using Mascot Version 2.2.04 (Matrix Science) with the following settings: 2 ppm and 0.8-Da deviation for precursor and fragment masses, respectively; no enzyme was specified. Mascot output files were loaded into Scaffold (http://www.proteomesoftware.com) and exported to Excel.

### HLA Class I Binding Prediction and Synthesis of Peptides

The capability of peptides to bind to which HLA-I molecules was predicted via the netMHC server (http://www.cbs.dtu.dk/services/NetMHC; [26], [27]), which makes use of approximation algorithms via artificial neural networks (ANNs) and is trained on 9- to 11-mer peptides to predict binding to HLA-I antigen binding pockets. Predicted peptides were synthesized by standard Fmoc chemistry and using a Syro II peptide synthesizer (MultiSyntech, Witten, Germany), as described previously [28]. The integrity of each peptide was routinely validated by HPLC and mass spectrometry.

### Competition-based Cellular Peptide Binding Assay

To test binding affinity of eluted and predicted peptides to HLA-I, competition-based cellular peptide binding assays were performed as described earlier [21]. In short, B-LCLs were treated with a mild acid (1∶1 mixture of 0.263M C_6_H_8_O_7_*H_2_O and 0.126M Na_2_HPO_4_*2H_2_O) for 1 min to remove the naturally HLA-I bound peptides. Cells were buffered with cold IMDM containing 2% FCS immediately thereafter and resuspended at a concentration of 4×10^5^ cells/ml in 2% FCS and 1.5 µg/ml human β2m (Sigma-Aldrich). Then, 4×10^4^ cells/well were incubated with 150 nM of fluorescently labeled reference peptide and a serial dilution of one of the eluted or predicted peptides. The following HLA-I allele-restricted reference peptides were used: FLPSDCFPSV (for HLA-A0201), KVFPCALINK (for HLA-A0301), APAPAPCWPL (for HLA-B0702) and GEFGGCGSV (for HLA-B4002), each containing a fluorescent label bound to the cysteine residue [21]. After overnight incubation at 4°C, cells were washed twice in PBS supplemented with 1% BSA and fixed in 0.5% paraformaldehyde. Cells were analyzed with CellQuestPro or FlowJo software (Tree Star, Ashland, OR, USA). IC50 values were defined with GraphPad Prism 4.02 (GraphPad Software Inc., La Jolla, USA) using the following formula: Y = Bmax*X/(IC50+X), in which Bmax is the maximal binding capacity of the positive control peptide, X the concentration of peptide tested and IC50 the concentration of peptide needed to reach half-maximal binding.

### Mouse Immunizations

HLA-A2 transgenic mice (B6 background HLA-A2/H2-D; [29]) were immunized subcutaneously with 50 µg of invariant chain peptides and 150 µg HBV T helper peptide (TPPAYRPPNAPIL) in PBS. The injection side was covered with 60 mg of Aldara cream containing 5% imiquimod (3 M Pharma Nederland BV). Immunization was repeated on day 7 combined with two intraperitoneal injections of 600.000 IU human recombinant IL-2 (Novartis) on day 7 and 8 [30]. The predicted invariant chain peptides were pooled to a total of 50 µg each. As positive control, mice were injected with 50 µg of MLIVYVRFWWL. At day 13, blood samples were taken and tested for CD8^+^ T cell reactivity by overnight incubation with the corresponding peptide and intracellular staining for IFN-γ.

## Results

### CLIP Presented on HLA-DR-negative Leukemic Cells from Patients is not Explained by Plasma Membrane Expression of HLA-II or CD74

**Figure 1:**
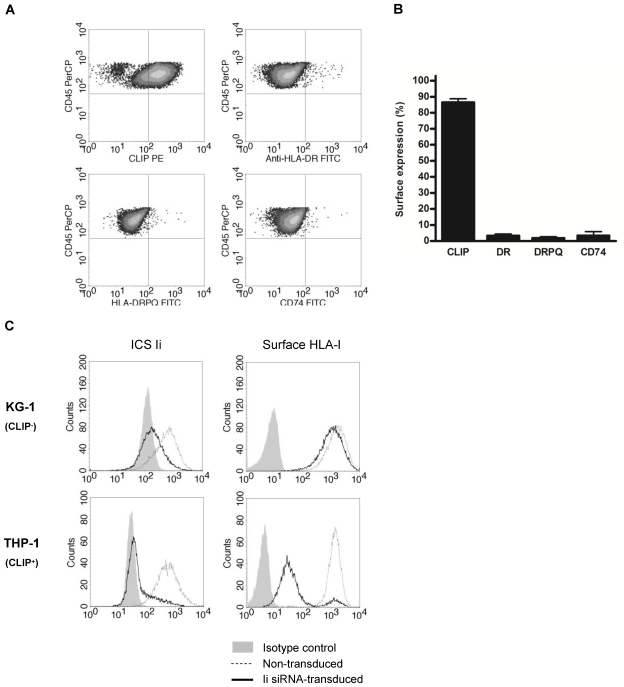
Surface display of the CLIP epitope on HLA-II-negative leukemic cells and influence of the invariant chain on HLA-I expression. (A) Surface expression of CLIP, HLA-DR, total HLA-II (‘HLA-DRPQ’) and invariant chain (‘CD74’) of myeloid cells from an acute promyelocytic leukemia (APL) patient, as determined by flow cytometry. Myeloid cells were defined as CD45^dim^/SSC^low/int^ and expression thresholds were set according to unstained myeloid cells. (B) Quantitative analysis on frequencies of myeloid cells from APL patients that express CLIP (n = 9), HLA-DR (n = 9), total HLA-II (n = 6) and CD74 (n = 6). Frequencies indicate percentage tumor cells that reach threshold expression based on unstained leukemic cells. (C) The effect of invariant chain Ii down-modulation in KG-1 (CLIP^-^) and THP-1 (CLIP^+^) leukemic cells on HLA-I expression at the cell surface. Intracellular staining (ICS) of Ii (PIN1.1) and surface staining of HLA-I (W6/32) were compared between Ii siRNA-transduced and non-transduced cells.

We found that patients with acute promyelocytic leukemia (APL; [31]), a genetically determined subtype of HLA-DR-negative acute myeloid leukemia (AML), contained a high frequency of leukemic cells with surface display of the CLIP epitope. Since CLIP expression was not observed on normal promyelocytes from healthy individuals, this remarkable observation may suggest that CLIP presentation at the plasma membrane of these leukemic cells is leukemia-specific and occurs in the context of other types of HLA-II proteins or as unprocessed Ii. To examine the underlying mechanism of CLIP presentation, we analyzed protein expression of total HLA-II and CD74 (*i.e.* unprocessed Ii) at the plasma membrane. Flow cytometric analysis however showed a lack of both proteins on HLA-DR^−^CLIP^+^ leukemic cells (Figure 1A and B), which shows that the expression of CLIP can neither be attributed to its presentation by HLA-DP or HLA-DQ molecules, nor to the expression of unprocessed Ii.

### Invariant Chain Silencing in Myeloid Leukemia Cell Lines Differentially Influences HLA-I Surface Expression

In the mutant TAP- and HLA-II-deficient T2 cell line, the precursor of CLIP, Ii, was shown to interact with H-2D^b^ alleles [32]. In addition, Ii can transport HLA-I molecules to endosomal compartments where exogenous peptides are present [33]. We next investigated whether there was a relation between Ii and HLA-I cell surface expression in CLIP-presenting and non-presenting leukemic cells. Retroviral introduction of Ii-specific siRNAs strongly downmodulates CLIP expression on several myeloid leukemia cell lines, as we recently reported [7]. When these cell lines were assessed for HLA-I protein expression by flow cytometry, KG-1 cells, which present low surface levels of CLIP [34], revealed hardly any effect of Ii silencing (Figure 1C). In THP-1 cells, which highly express CLIP [34], however, silencing of Ii induced a strong reduction in HLA-I surface expression (Figure 1C). In addition, we found indications that Ii was indeed able to associate with HLA-I-related products in leukemic cells, since Ii isoforms were present in HLA-I and Ii immunoprecipitates (Figure S1A), respectively. This was mostly seen in Ii-overexpressing Kasumi-1 cells (Figure S1A and S2), which also showed an increase in free-form β2m after Ii silencing (Figure S1B). The involvement of Ii in HLA-I plasma membrane expression in CLIP-presenting leukemic cells suggests a relation between surface expression of CLIP and HLA-I molecules. Also TAP expression was limited in CLIP^+^ THP-1 cells, in contrast to CLIP^–^ KG-1 cells (Figure S2), indicating that the involvement may rely on the ability to process endogenous antigens for loading in the ER.

### Peptides Derived from the Invariant Chain are Presented at the Cell Surface by HLA-I Molecules

**Figure 2:**
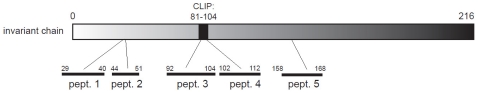
Invariant chain-derived peptides identified in isolated HLA-I molecules of B-LCLs. Peptide elutions of purified HLA-I molecules from EBV-transformed B-LCLs resulted in the identification of five peptides originating from the invariant chain. HLA-I purification and subsequent mass spectrometry analysis are described in *Materials and Methods*. Of note, peptide 3 and 4 are located in the CLIP region, known for universal binding to HLA-II molecules.

We next assessed whether HLA-I molecules can indeed present Ii-derived peptides at the plasma membrane. The peptide repertoires of HLA-I molecules on EBV-transformed B cells, including CLIP-positive JY cells (Figure S3) were evaluated through biochemical purification. Out of the total characterized peptide repertoire, five peptides were derived from the Ii protein (Figure 2 and Table 1), the sequences of which were validated by mass spectrometry according to their synthetic counterparts. Remarkably, two peptides were located within or near the CLIP region, known for its association with HLA-II peptide-binding grooves. Isolation of the 5 identified peptides was restricted to HLA-A0201 and HLA-B0702 alleles presented on EBV-transformed B cells. To examine the HLA-I allele specificity and binding affinity of these peptides, competition-based peptide binding assays were performed (Table 1). Four out of five peptides had binding capacity to the B0702 allele and three of these were even categorized as high affinity binders. Notably, peptide 1 and peptide 3, the peptide located within the CLIP region, also bound to the A0201 allele, which might suggest a promiscuous binding capacity of these peptides to HLA-I. Altogether, these data demonstrate that surface HLA-I molecules are able to present a variety of peptides generated from Ii.

**Table 1:** HLA-I binding affinity of eluted peptides derived from the invariant chain.

HLA-I allele		HLA-A201		HLA-B0702	
*Peptide no.* *	*Peptide sequence*	*IC50* †	*Affinity* ‡	*IC50*	*Affinity*
1	SRGALYTGFSIL	10.89	Int	1.39	High
2	LLAGQATT	>100	–	40.85	Low
3	RMATPLLMQALPM	13.66	Int	0.36	High
4	LPMGALPQGPM	>100	–	0.41	High
5	ETIDWKVFESW	>100	–	>100	–

* See Figure 2 for amino acid position in the invariant chain protein.

† IC50 is the concentration used to obtain half maximal competition and represents the mean value of two independent experiments.

‡ Binding affinity is classified according to the following IC50 cut-off values: high affinity, ≤5 µM; intermediate (int) affinity, 5–15 µM; low affinity, 15–100 µM; no binding, >100 µM [21].

### Invariant Chain-derived Peptides Located at the CLIP Region Show Promiscuous Binding to Various HLA-I Alleles

**Figure 3:**
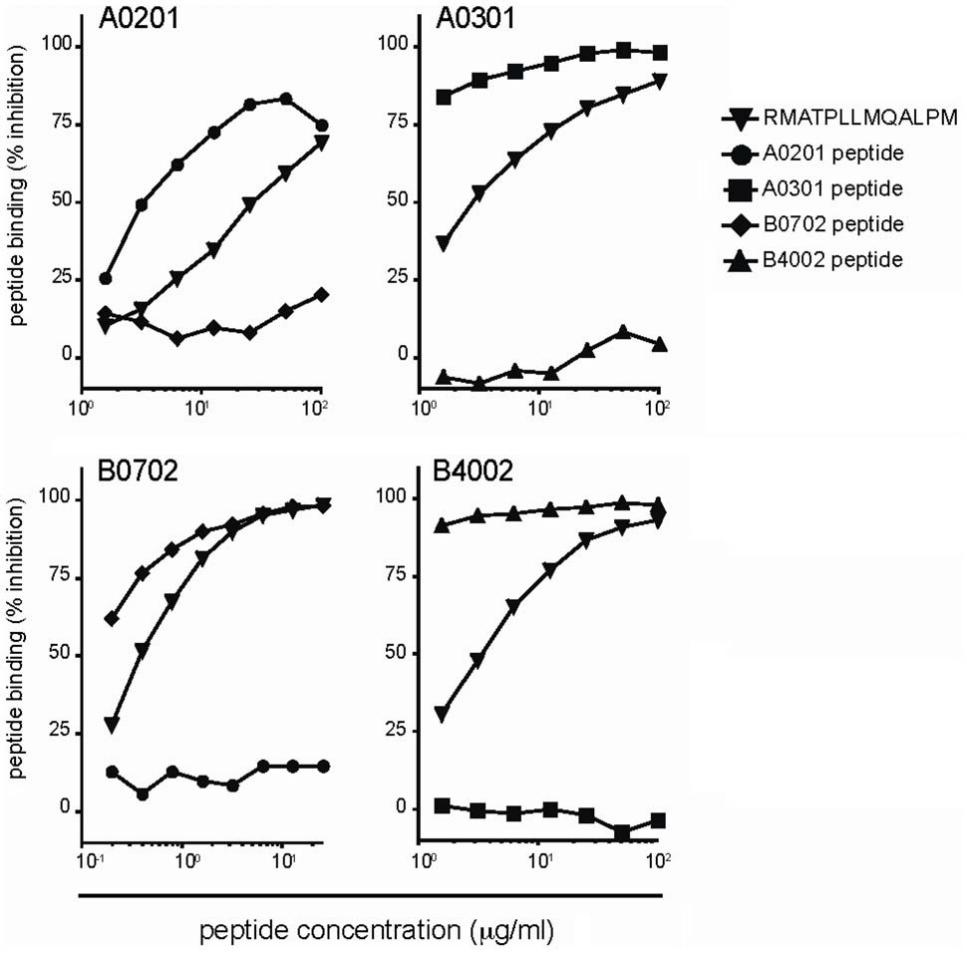
Promiscuous binding of CLIP-peptide to distinct HLA-I alleles, representing four supertypes. The CLIP peptide RMATPLLMQALPM (peptide 3) was tested for binding affinity in a competition-based cellular peptide binding assay. The four tested HLA-I alleles (HLA-A0201, -A0301, -B0702 and -B4002) harbor a completely distinct binding pocket and bind different peptide ligands. CLIP peptide shows intermediate to high binding affinity to all of these. Separate positive control peptides efficiently bind to their respective HLA allele: GILGFVFTL (A0201 peptide), QVPLRPMTYK (A0301 peptide), SPSVDKARAEL (B0702 peptide) and GEFGGFGSV (B4002 peptide) with IC50 values of 3.7, 0.2, 0.7 and 0.2, respectively [21]. The peptide concentration started at 100 µg/ml for HLA-A0201, -A0301 and -B4002 and 25 µg/ml for HLA-B0702, followed by a serial dilution of a factor two. Exact IC50 values of the CLIP peptides are depicted in Table 2.

As CLIP is known to bind promiscuously to the binding groove of a broad range of HLA-II molecules [35], we further explored the HLA-I binding specificity of the eluted peptide located at the CLIP region of Ii (peptide no. 3; Figure 2 and Table 1). This naturally presented peptide (RMATPLLMQALPM) demonstrated a high affinity for both HLA-A0201 and -B0702 (Table 1). To determine if peptide RMATPLLMQALPM could bind promiscuously to HLA-I molecules, we addressed its binding capacity to HLA-I molecules bearing structurally different binding grooves. HLA-I molecules are classified according to overlapping binding repertoires and consensus structures in the main peptide binding pockets, so-called supertypes [36]. HLA-A0201 and -B0702 are well known representatives of the A2 and B7 supertype, respectively. The same accounts for HLA-A0301 and -B4002, which represent the common supertypes A3 and B40 [36]. Interestingly, peptide RMATPLLMQALPM bound with a relatively high affinity to all four HLA-I alleles (Figure 3), which have completely different peptide-binding grooves, suggesting that the CLIP sequence involved in promiscuous HLA-II binding also underlies a promiscuous binding to HLA-I molecules.

The unexpected promiscuous binding of peptide RMATPLLMQALPM to distinct HLA-I molecules prompted us to systematically assess length variants of peptides located in the CLIP region of Ii for such binding feature. The CLIP amino acid sequence of Ii was subjected to standard HLA-I binding prediction algorithms for 9- to 11–mer peptides [26], [27], which resulted in highly predictive values of three length variants (9-, 10- and 11-mer) of the naturally presented 13-mer RMATPLLMQALPM (Table 2). The three peptides were predicted to selectively bind to HLA-A2 subtypes and, in case of the 11-mer, also to -B7 subtypes. Subsequently, we tested these predicted peptides for actual binding to our selected alleles representing four different supertypes, HLA-A0201, -B0702, -A0301 and -B4002. In contrast to their predictions, all the length variants bound to each of the four HLA-I alleles with reasonably high affinity (Table 2), indicating that peptides located in the CLIP region of Ii have universal binding abilities to polymorphic HLA-I molecules. Finally, we tested whether the identified Ii-derived peptides were immunogenic in the context of HLA-I, as reported for self peptides termed T-cell epitopes associated with impaired peptide processing (TEIPPs; [37]–[39]), but immunizations of HLA-A2 transgenic mice with these peptides showed no CD8^+^ CTL responses (Figure S4). From these data, we conclude that the CLIP region of Ii harbors a unique peptide sequence that can accommodate binding to structurally completely different grooves of both HLA-I and -II.

**Table 2:** HLA-I binding affinity of peptide length variants located in the CLIP region of the invariant chain.

HLA-I allele			HLA-A201		HLA-B0702		HLA-A0301		HLA-B4002	
*Peptide sequence*	*Position* †	*Predicted allele* ‡	*IC50* §	*Affinity* ¶	*IC50*	*Affinity*	*IC50*	*Affinity*	*IC50*	*Affinity*
RMATPLLMQALPM*	92–104	NA	13.81	Int	0.36	High	3.35	High	3.43	High
KMRMATPLL	90–98	A0201	10.90	Int	0.47	High	6.43	Int	0.44	High
RMATPLLMQA	92–101	A0201	8.08	Int	7.75	Int	2.45	High	2.05	High
RMATPLLMQAL	92–102	A0201+ B0702	4.49	High	1.85	High	4.28	High	0.48	High

* Peptide found with HLA-I elutions.

† Amino acid (AA) position in the invariant chain protein.

‡ Predicted HLA-I allele to which the peptide binds. Peptide binding prediction was done with netMHC (http://www.cbs.dtu.dk/services/NetMHC). Binding predictions can be made for peptide lengths between 8 and 11 for all alleles with a novel approximation algorithm using artificial neural networks trained on 9-mer peptides [26], [27]. Only peptides are shown with a predicted binding affinity of 500 nM or stronger.

§ IC50 is the concentration used to obtain half maximal competition and represents the mean value of two independent experiments.

¶ Binding affinity is classified according to the following IC50 cut-off values: high affinity, ≤5 µM; intermediate (int) affinity, 5–15 µM; low affinity, 15–100 µM; no binding, >100 µM [21].

## Discussion

Processing and presentation of antigens via HLA molecules by APCs is the key mechanism for generating a specific immune response against pathogens and TAAs. In normal APCs, Ii and CLIP have essential roles in the HLA-II antigen presentation pathway. We and others previously described that in tumor cells that are able to function as APC, expression of Ii and CLIP could serve as immune escape mechanism by interference with HLA-II-mediated TAA presentation [5], [8], [40]. Here, we reveal that Ii can also be associated with HLA-I antigen presentation in malignant cells. This study points at an alternative HLA-I antigen presentation pathway that may affect current concepts of antigen cross-presentation and tumor immune escape.

One of the early events during HLA-I processing is the binding of heavy chains to β2m in the ER. This results in HLA-I stabilization, appropriate folding and incorporation into the class I peptide-loading complex, consisting of TAP and other ER-resident chaperones important for efficient HLA-I peptide loading. Our observation that Ii is involved in HLA-I processing in leukemic cells (Figure 1C and Figure S1) indicates that it may take part of this process as well. This agrees with earlier studies using HLA-I-transfected T2 cells [32], [41], [42] and suggests that like newly synthesized HLA-II αβ complexes, also HLA-I/β2m heterodimers can interact with Ii in the ER lumen until peptides are loaded. As Ii inhibits premature peptide binding to HLA-II complexes in the ER [10], the question rises how the association of HLA-I with Ii is related to the function of the class I peptide-loading complex in this compartment. Ii-silenced THP-1 cells lacking the TAP molecule (Figure S2) revealed a strong reduction in HLA-I surface expression level, in contrast to TAP-expressing KG-1 cells (Figure 1C). Additionally, in transfected TAP-deficient T2 cells, HLA-I/Ii complexes relied on the absence of TAP-dependent HLA-I peptides for their stability [32], [41] and were also part of the class I peptide-loading complex [43]. For this, we propose that two different types of competition mechanisms can be active in APCs: one involving the binding of Ii to either HLA-I or HLA-II and one involving the binding of either Ii or peptide loading complex peptides to HLA. In this model, the balance between TAP and Ii expression might be of major importance. The likelihood of Ii to interact with HLA-I molecules increases in the absence of TAP, as has been described for T2 cells [43], but likely also in the situation of Ii abundance, as we demonstrate for Ii-high Kasumi-1 cells (Figure S1 and S2). Our finding that peptides derived from Ii were predicted and able to bind to HLA-A0201 and -B0702 (Table 1 and Figure 2) further suggests that the peptide-binding groove is the HLA-I site with which Ii associates. Indeed, mutations in the binding groove of HLA-B7 molecules affected their interaction with Ii and expression at the plasma membrane in transfected T2 cells [42]. It remains to be determined which specific HLA-I alleles are prone to bind to Ii, but the promiscuous binding of CLIP-related Ii peptides to the four tested HLA-I superfamilies (Table 2 and Figure 3) indicates great similarity to the promiscuous binding capacity of the CLIP region in the context of HLA-II [44]. However, this contradicts with the large differences in key anchor motifs between each HLA-I superfamily. The shallow binding grooves of HLA-II binds peptides based on favorable interactions rather than need of specific amino acids at each position. Even one amino acid with high affinity to the HLA-II binding groove is sufficient for peptide binding, which could also be the case for HLA-I, as the methionine at position 99 of the CLIP region served as crucial anchor residue for H2-Kb molecules [43].

The observed involvement of Ii and CLIP in HLA-I processing in leukemic cells, as the result of a potentially imbalanced TAP/Ii ratio, can have different outcomes on tumor immune escape. In Ii overexpressing leukemias, such as AML (*e.g.* Kasumi-1 cells; Figure S2) and chronic lymphocytic leukemia (CLL; [45]), but also colorectal carcinoma [46], Ii instead of TAA-derived peptides may be bound to HLA-I molecules after synthesis in the ER, thereby preventing TAA presentation at the plasma membrane and recognition by TAA-specific CTLs. In TAP-deficient tumors, self peptides termed TEIPPs have been identified that are specifically detected by CTLs [37], [39]. In addition, we observed a possible relation of TAP and CLIP expression in leukemia, with CLIP^-^ leukemic cells positive and CLIP^+^ leukemic cells negative for TAP (including APL cells in Figure 1A; Figure S2; [6]). Therefore, the formation of HLA-I/Ii complexes may lead to processing of Ii into CLIP and surface presentation of this self-peptide as a TEIPP. In immunized HLA-A2 transgenic mice however, we could not detect CTL responses against CLIP-related Ii peptides (Figure S4). Another possibility is that HLA-I/Ii complexes in leukemic cells are transported from the ER into the endo-lysosomal pathway to be loaded with TAA-derived peptides obtained from authophagy and lysosomal processing. In normal APCs, Ii is classically known for its role in trafficking HLA-II molecules to the MIICs. It was recently demonstrated that in DCs Ii is also able to direct CD70 molecules to these compartments [20], indicating that the interaction of Ii with HLA-I molecules could account for such a transporting function as well and represent a novel cross-presentation pathway. Sugita and colleagues already demonstrated a role for Ii in the transport of HLA-I molecules to endosomes [33], which might explain the accumulation of newly synthesized HLA-I molecules in endosomal storage compartments [13]. Indeed very recently, in murine DCs, Ii was shown to be critical in MHC class I trafficking from the ER to late endosomes for antigen loading, serving as a mediator of cross-presentation [47]. Although further HLA-I/Ii processing for peptide loading in such endosomal compartments remains undefined, we detected CLIP-related Ii peptides in HLA-I-specific eluates of B-LCLs (Table 1 and Figure 2), indicating that Ii can be processed to CLIP in HLA-I. These peptides were not derived from HLA-II molecules, as validations for contamination with HLA-II during each step of peptide elution showed HLA-I heavy chains and β2m, but no HLA-II monomers. It is thus appealing to further examine HLA-I-mediated exchange of CLIP for antigenic peptides in the endo-lysosomal pathway as well as presentation of CLIP at the plasma membrane for the effect on CTL activation.

In this report, we present important data showing the promiscuous involvement of Ii and CLIP in the HLA-I antigen presentation pathway of leukemic APCs. To define the similarity with HLA-II processing, further exploration of their role in intracellular transport and peptide loading of HLA-I molecules is necessary. Since Ii and CLIP are involved in both HLA-I and HLA-II antigen presentation, it will be attractive to design immunotherapeutic strategies that modulate their expression, thereby controlling antigen presentation with the purpose to target immune surveillance against leukemias and possibly prevent autoimmunity.

## Supporting Information

File S1 **Supplementary methods.**
(PDF)

Figure S1 **Association of Ii with the HLA-I complex in leukemic cells.** (A) Immunoblotting of Ii in both HLA-I (W6/32) and Ii (PIN1.1) immunoprecipitates of KG-1, THP-1 and Kasumi-1 cells. IgG immunoprecipiates were used as negative controls. (B) The presence of free-form β2m (12 kD) in total lysates of leukemic cells derived from the Ii-overexpressing Kasumi-1 cell line. Immunoblots for Ii and β2m were performed under SDS conditions and antibodies used for staining were against Ii (PIN1.1) or β2m (rabbit polyclonal antibody, kindly provided by Dr. J.J. Neefjes, NKI, Amsterdam, The Netherlands).
(TIFF)

Figure S2 **Immunoblot analysis of Ii and TAP expression in the KG-1 (CLIP^−^), THP-1 (CLIP^+^) and Kasumi-1 (CLIP^+^) leukemic cell line.** Blots were loaded with total cell lysates and stained with primary anti-Ii (clone PIN1.1) and anti-TAP1 (clone 148.3, a kind gift from Dr. E.J. Wiertz and Dr. M.E. Ressing, University Medical Center Utrecht, The Netherlands) MoAb, demonstrating specific bands of 33 kD and 74 kD, respectively.
(TIFF)

Figure S3 **CLIP expression on the surface of T2 and EBV-transformed JY cells, as determined by flow cytometry using a PE-labeled cerCLIP.1 MoAb.**
(TIFF)

Figure S4 **The** ***in vivo*** **effect of Ii-derived peptides on CD8^+^ T cell activation.** HLA-A2 transgenic mice (n = 3 per group) were immunized with Ii-derived peptides identified in peptide elution studies (see Table 1 for numbering), or with a pool of Ii-derived peptides identified on basis of an *in silico* prediction algorithm (see Table 2). After 13 days, peptide-reactive CD8^+^ T cells from blood were stained with PE-labeled CD8 and APC-labeled anti-IFN-γ antibodies and analyzed by flow cytometry. IFN-γ-positive CD8^+^ T cell frequencies are expressed as the percentage within the total pool of CD8^+^ T cells.
(TIFF)
