# Supplementary material for: Promiscuous Binding of Invariant Chain-Derived CLIP Peptide to Distinct HLA-I Molecules Revealed in Leukemic Cells
Source: PLoS One. 2012 Apr 26;7(4):e34649. doi: 10.1371/journal.pone.0034649 (PMC3338516; doi:10.1371/journal.pone.0034649)
Supplement: File S1 — Supplementary methods. (PDF) [file pone.0034649.s001.pdf]

For co-immunoprecipitation, 10-20 x 10^6 snap-frozen cells were dissolved in ice-cold lysis buffer, consisting of PBS with 1% Ipegal and 15% protease inhibitor cocktail [Complete, 1 tablet/7.5 ml H_2O; Boehringer Mannheim, Mannheim, Germany]). Cell remnants and nuclei were removed by centrifugation (5 min, 10,000 g) and protein content was assessed by the Bio-Rad protein assay (Biorad Laboratories, Hercules, CA, USA). Equal amounts of whole cell lysate (~0.5 mg) per sample were precleared using 1.0 µg/ml mouse IgG_1 and 20 µl/ml Protein G PLUS-Agarose reagent (Santa Cruz Biotechnology) for 30 min. Proteins were immunoprecipitated overnight with specific MoAb and Protein G PLUS-Agarose reagent. Immunoprecipitates were washed four times with PBS and taken up in reducing electrophoresis sample buffer.

Western blotting was performed by electrophoresis of samples on 12.5% polyacrylamide gels with SDS and their subsequent transfer onto methanol-activated PVDF membranes. Different protein amounts from whole cell lysates were used for loading on gel: 20 µg for Ii and 70 µg for TAP1. Membranes were pre-incubated for 1 h at 4°C in blocking buffer (5% blotting grade nonfat milk [Biorad]) in TBS-T; 10 mM Tris-HCl, pH 8.0, 0.15 M NaCl, 0.1% Tween-20) and incubated overnight with MoAbs. After washing with TBS-T (4x), membranes were incubated for 1 h with HRP-conjugated goat anti-mouse antibody (Dako). TrueBlot™ ULTRA anti-mouse IgG (eBioscience, San Diego, CA, USA) was used to limit the detection of immunoprecipitated heavy and light chains. Protein complexes were visualized using Amersham ECL Western Blotting Detection Reagents (GE Healthcare, Buckinghamshire, UK).
