# Supplementary figures and images for: Promiscuous Binding of Invariant Chain-Derived CLIP Peptide to Distinct HLA-I Molecules Revealed in Leukemic Cells
Source: PLoS One. 2012 Apr 26;7(4):e34649. doi: 10.1371/journal.pone.0034649 (PMC3338516; doi:10.1371/journal.pone.0034649)

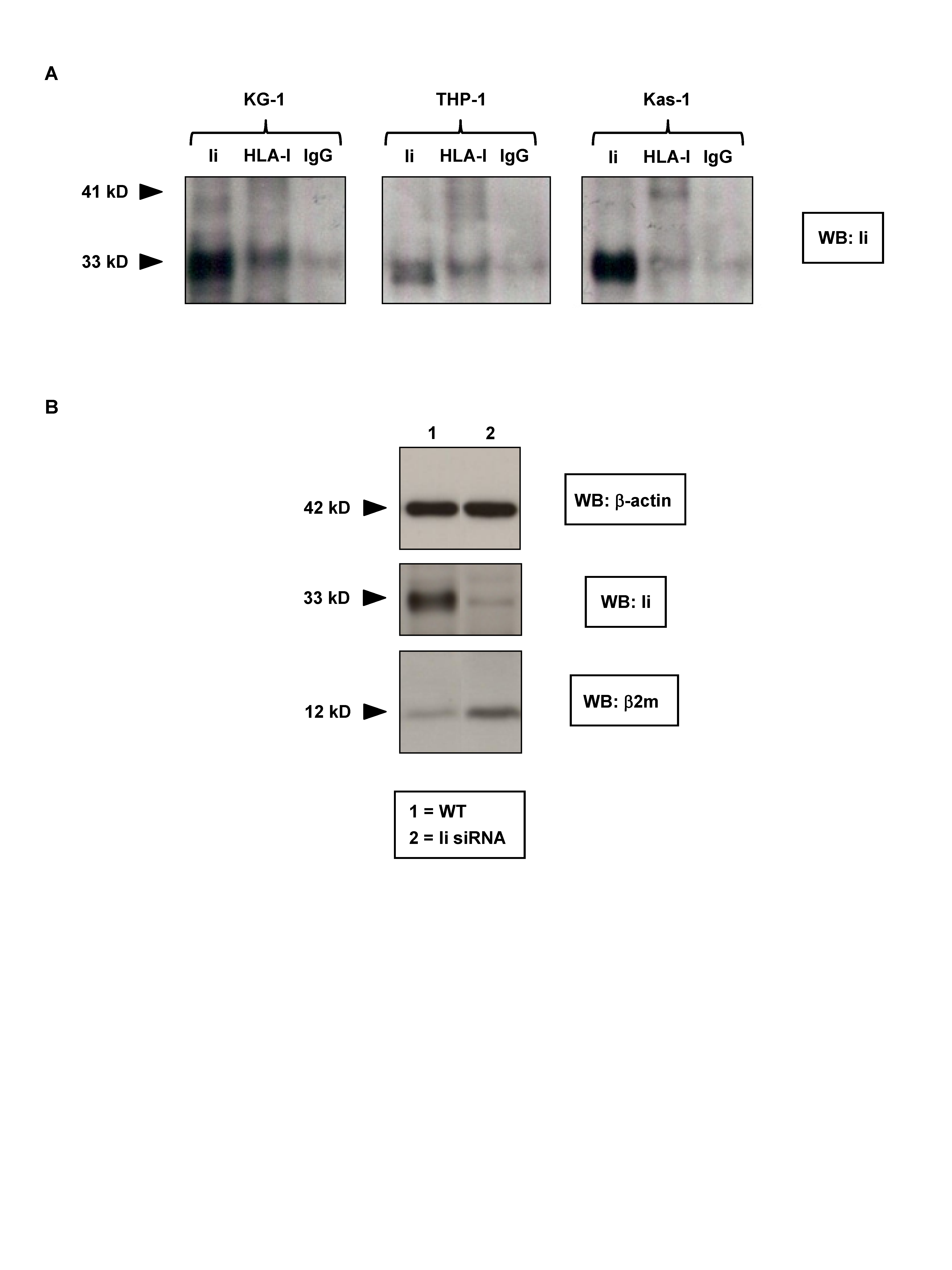

Supplement: Figure S1 — Association of Ii with the HLA-I complex in leukemic cells. (A) Immunoblotting of Ii in both HLA-I (W6/32) and Ii (PIN1.1) immunoprecipitates of KG-1, THP-1 and Kasumi-1 cells. IgG immunoprecipiates were used as negative controls. (B) The presence of free-form β2m (12 kD) in total lysates of leukemic cells derived from the Ii-overexpressing Kasumi-1 cell line. Immunoblots for Ii and β2m were performed under SDS conditions and antibodies used for staining were against Ii (PIN1.1) or β2m (rabbit polyclonal antibody, kindly provided by Dr. J.J. Neefjes, NKI, Amsterdam, The Netherlands). (TIFF) [file pone.0034649.s002.tif]

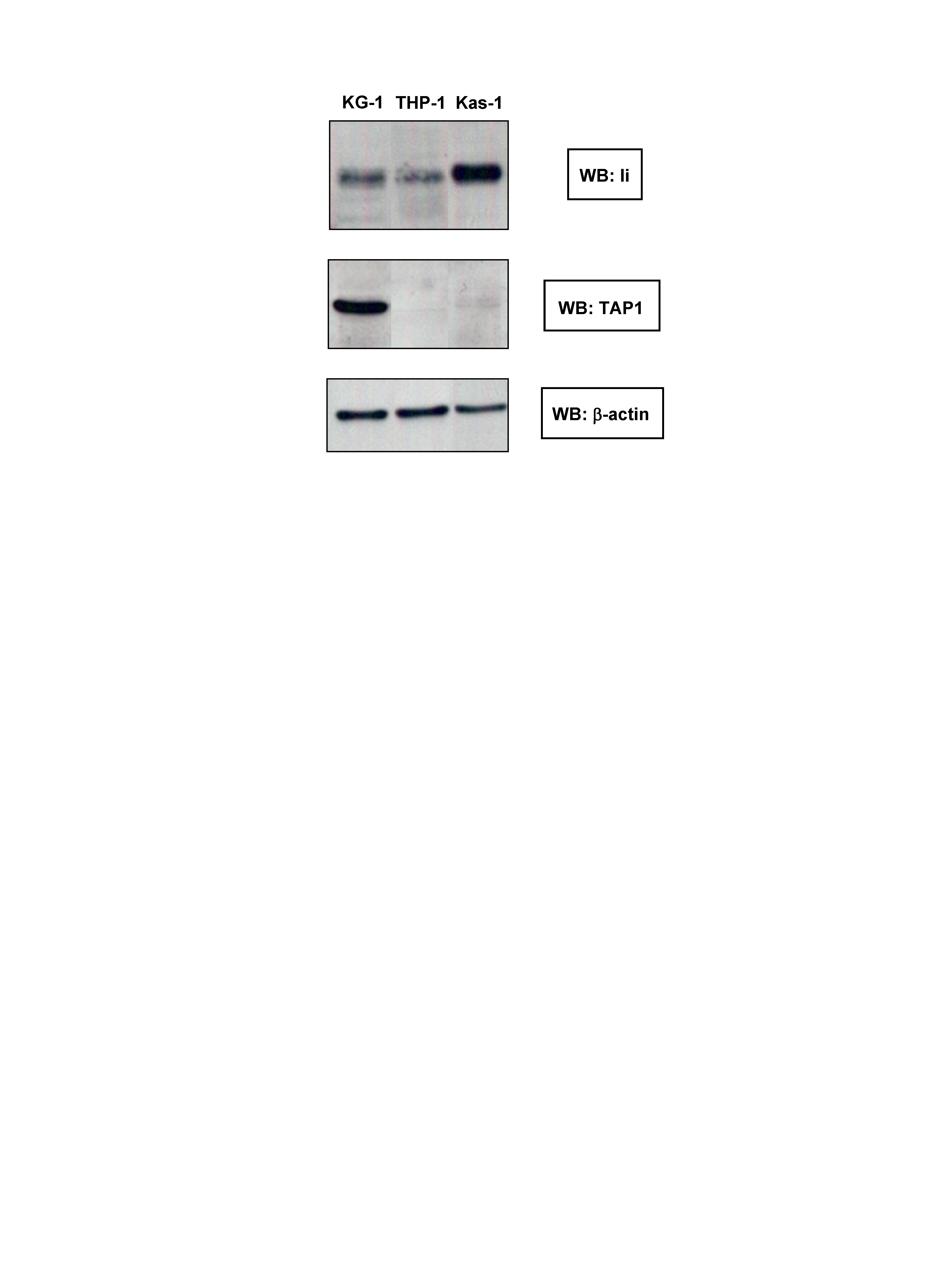

Supplement: Figure S2 — Immunoblot analysis of Ii and TAP expression in the KG-1 (CLIP−), THP-1 (CLIP+) and Kasumi-1 (CLIP+) leukemic cell line. Blots were loaded with total cell lysates and stained with primary anti-Ii (clone PIN1.1) and anti-TAP1 (clone 148.3, a kind gift from Dr. E.J. Wiertz and Dr. M.E. Ressing, University Medical Center Utrecht, The Netherlands) MoAb, demonstrating specific bands of 33 kD and 74 kD, respectively. (TIFF) [file pone.0034649.s003.tif]

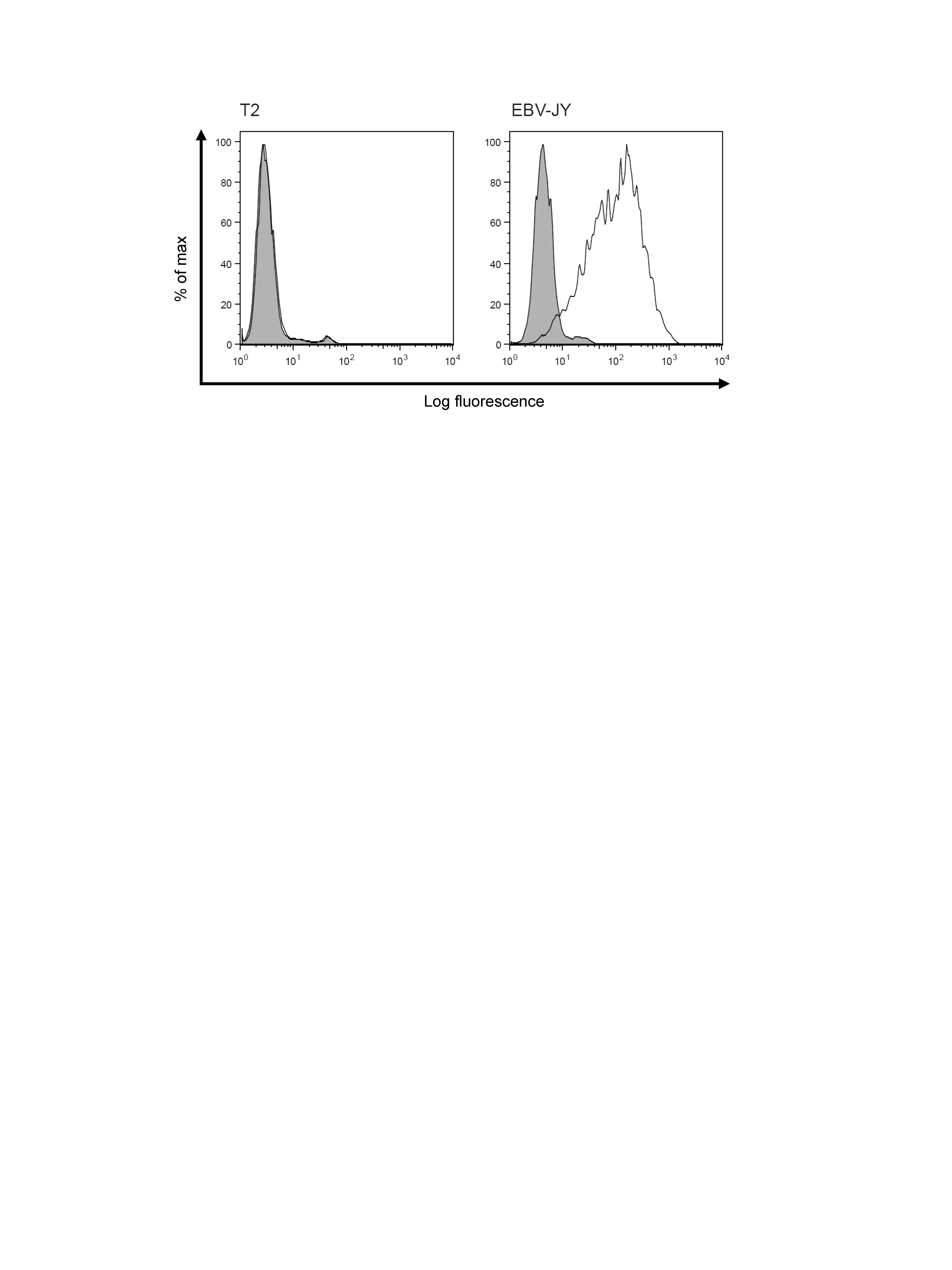

Supplement: Figure S3 — CLIP expression on the surface of T2 and EBV-transformed JY cells, as determined by flow cytometry using a PE-labeled cerCLIP.1 MoAb. (TIFF) [file pone.0034649.s004.tif]

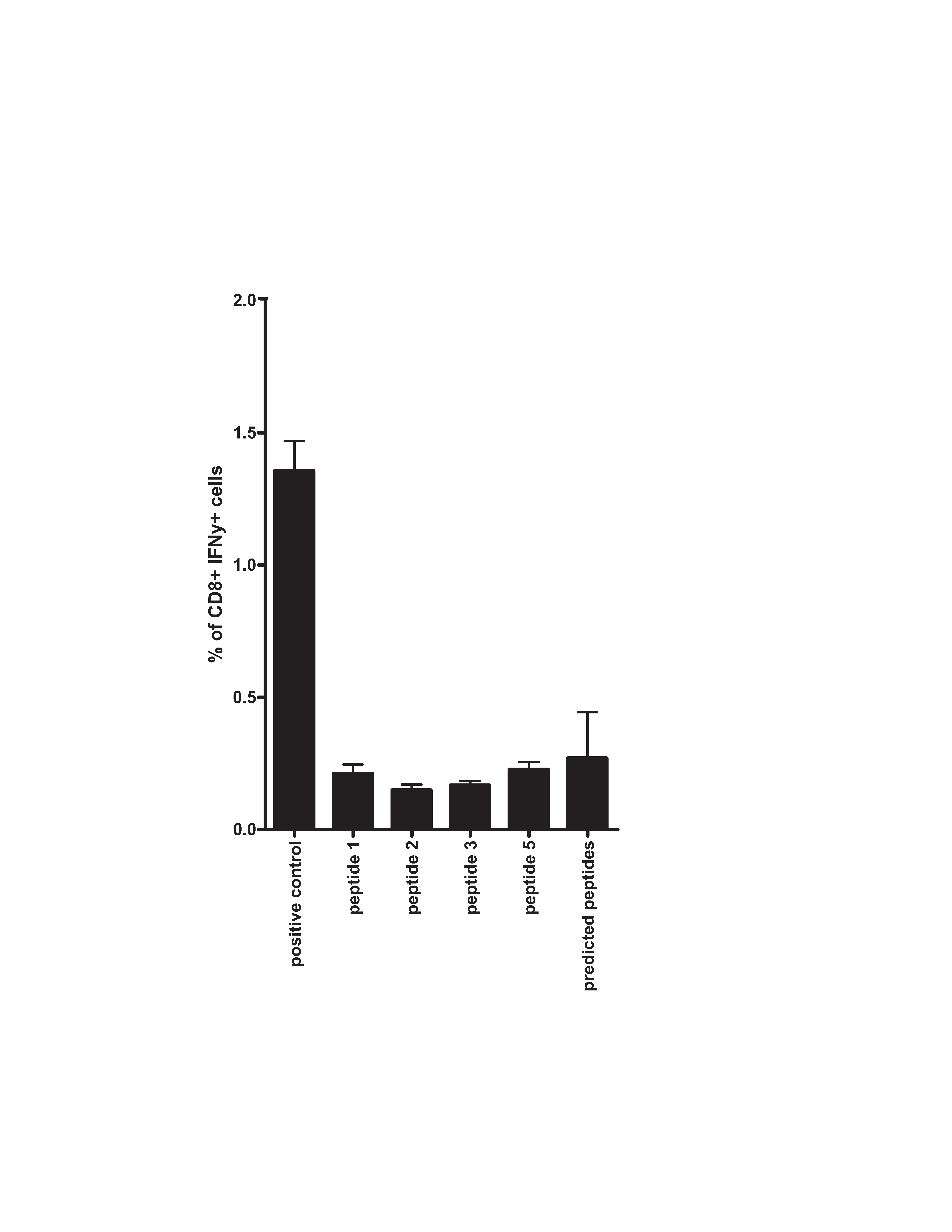

Supplement: Figure S4 — The in vivo effect of Ii-derived peptides on CD8+ T cell activation. HLA-A2 transgenic mice (n = 3 per group) were immunized with Ii-derived peptides identified in peptide elution studies (see Table 1 for numbering), or with a pool of Ii-derived peptides identified on basis of an in silico prediction algorithm (see Table 2). After 13 days, peptide-reactive CD8+ T cells from blood were stained with PE-labeled CD8 and APC-labeled anti-IFN-γ antibodies and analyzed by flow cytometry. IFN-γ-positive CD8+ T cell frequencies are expressed as the percentage within the total pool of CD8+ T cells. (TIFF) [file pone.0034649.s005.tif]
